# Caffeic Acid and Erythromycin: Antibacterial and Synergistic Effects on Staphylococci

**DOI:** 10.3390/ph18070964

**Published:** 2025-06-26

**Authors:** Małgorzata Kępa, Maria Miklasińska-Majdanik, Aleksandra Haczyk, Arkadiusz Matuła, Robert D. Wojtyczka

**Affiliations:** Department of Microbiology, Faculty of Pharmaceutical Sciences in Sosnowiec, Medical University of Silesia in Katowice, Jagiellońska 4, 41-200 Sosnowiec, Poland; mmiklasinska@sum.edu.pl (M.M.-M.); aleksandra.haczyk.96@gmail.com (A.H.); arekmatula1997@gmail.com (A.M.); rwojtyczka@sum.edu.pl (R.D.W.)

**Keywords:** natural compounds, fractional inhibitory concentration, multidrug-resistant strains, half maximal inhibitory concentration

## Abstract

**Background:** Antibiotic-resistant bacteria, especially *Staphylococcus* species, are a growing concern in healthcare settings and infections caused by multidrug-resistant strains are difficult to treat. Therefore, it is imperative to explore new treatment methods for these infections such as combinations of natural compounds with antibiotics. **Methods:** The main objective of this study was to investigate the antimicrobial activity of caffeic acid against staphylococcal strains. The viability of bacterial cells and half maximal inhibitory concentration (IC_50_) for caffeic acid were also examined. The minimum inhibitory concentration (MIC) of the caffeic acid was determined using a serial microdilution method. To study the combined effect of caffeic acid and erythromycin, the fractional inhibitory concentrations (FICs) were determined. **Results:** Caffeic acid inhibited the growth of all the tested isolates, with MIC values ranging from 256 to 1024 µg/mL and reduced bacterial cell viability at concentrations corresponding to MIC values. Caffeic acid and erythromycin showed a synergistic effect when used together against three examined strains and had an additive effect against two isolates. However, their combination was indifferent against the seven remaining staphylococci tested. **Conclusions:** The results of our research demonstrate that caffeic acid has antimicrobial properties against the tested strains.

## 1. Introduction

Nowadays, the main etiological factors of nosocomial infections are coagulase-negative staphylococci, with *Staphylococcus epidermidis* (*S. epidermidis*) being the most dominant. Infections caused by *Staphylococcus aureus* (*S. aureus*) are an equally serious problem. These bacteria naturally colonize human skin and mucous membranes, forming the microbiota of this environment. However, if tissue continuity is disrupted, e.g., during the medical procedures, these staphylococci can cause difficult-to-treat infections. Especially dangerous are methicillin-resistant staphylococcal strains [1]. *Staphylococcus aureus* causes diseases which range from skin and soft tissue infections, such as folliculitis or urinary tract infections, to more serious conditions such as sepsis, pneumonia, or osteitis [2,3]. *S. aureus* has been added by the World Health Organization (WHO) to the list of priority pathogens resistant to antibiotics [4]. *S. epidermidis* poses a particular danger in immunocompromised patients and those undergoing invasive medical procedures. Moreover, *S. epidermidis* is a common cause of catheter or prosthesis infections due to its ability to form a biofilm that facilitates the colonization of medical implants and increases the risk of infection. Staphylococcal wound and burn infections, which are difficult to treat, are another major problem [5]. Therefore, the clinical strains selected for this study were isolated from wounds, including postoperative ones, in the case of *S. aureus*, and from blood and pericardial fluid in the case of *S. epidermidis*, to initially assess caffeic acid’s usefulness as an antibacterial agent applied topically to wounds or systemically.

The increasing antibiotic resistance of bacteria is a serious problem for public health. It is estimated that at least 700,000 people die due to infections caused by drug-resistant pathogens every year and this number may increase to 10 million by 2050, which will make these this one of the main causes of death in the world, overtaking cancer [6]. Many infections caused by drug-resistant bacteria do not respond to standard treatment regimens, even to “last chance” antibiotics [7]. Therefore, it is crucial to explore alternative treatments for such infections. The substances obtained from medicinal plants, which have antimicrobial properties, may support the action of commonly used antibiotics and could be such an alternative. Since natural compounds do not have sufficient antibacterial properties to be used as monotherapy, research in combination with antibiotics seems justified. Natural compounds can increase the potential of antibiotics by improving their pharmacokinetic and pharmacodynamic properties, lead to a reduction in the doses of antibiotics used, and thus reduce side effects. Moreover, natural compounds could influence the mechanisms of bacterial resistance. All the above aspects can contribute to inhibiting the spread of resistance among bacteria [8]. However, the cytotoxicity of natural compounds is an important aspect in the context of their use as antibacterial agents. The effects on eukaryotic cells should be determined to choose the right dosage and avoid side effects. In summary, studying the antibacterial properties of natural compounds may prove to be a way to combat the spread of antibiotic resistance among bacteria. However, all pros and cons should be considered to achieve an antibacterial effect without harming the patient [9,10].

**Figure 1:**
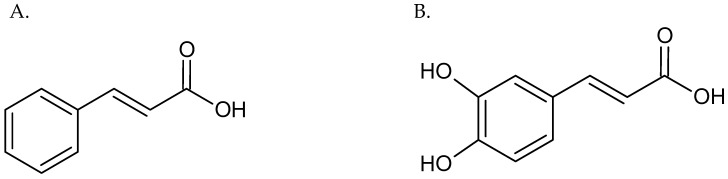
The structural formula of cinnamic acid (**A**) and caffeic acid (**B**).

Special attention in this aspect is paid to hydroxycinnamic acids, which belong to polyphenolic compounds. The characteristic structure of these compounds is a phenol ring with a radical containing a carboxyl group. Phenolic compounds differ from each other in the substituents on the phenol ring. Naturally occurring hydroxycinnamic acids are present in the cell wall of plants. Caffeic acid, or 3,4-dihydroxycinnamic acid, is a main representative belonging to phenolic compounds [11]. Caffeic acid is synthesized by plants as a secondary metabolite of chlorogenic acid and is a cinnamic acid derivative (Figure 1). Caffeic acid may be a structural part of monomers as organic acids, amides, and sugar esters, or, in the form of dimers, trimers, or flavonoid derivatives, it can also be combined with proteins or other polymers in the plant cell wall [12].

Caffeic acid can be found in coffee beans, tea leaves, oats, rice, fruit, argan oil, or olive food [11,13]. Many in vitro—and some in vivo—studies have demonstrated numerous biological properties of caffeic acid such as antioxidant, anti-inflammatory, antibacterial, antiviral, antiatherosclerosis, anticancer, immunomodulatory, antidiabetic, cardioprotective, and hepatoprotective effects. The above properties of caffeic acid are related to its ability to modulate inflammatory and oxidative processes. Its antioxidant properties are based on the neutralization of reactive oxygen species. Since oxidative stress is the cause of many diseases, the neutralization of free radicals could help to fight them [14,15,16,17,18,19,20,21,22,23].

Our previous work on caffeic acid’s antibacterial action was a pilot study aimed at selecting the most promising caffeic acid–antibiotic combination for further research. The antibacterial properties of caffeic acid against both reference and clinical strains of *S. aureus* have been demonstrated [13]. Furthermore, a reduction in the minimal inhibitory concentrations of erythromycin in the presence of caffeic acid was observed. *S. aureus* strains were treated with caffeic acid in combination with antibiotics, such as erythromycin, clindamycin, cefoxitin, and vancomycin. Interactions between caffeic acid and antibiotics were noted in 16 isolates exposed to a “caffeic acid–erythromycin” combination. In contrast, interactions of caffeic acid with other antibiotics were observed in fewer strains. The changes in MIC values of erythromycin after caffeic acid addition were statistically significant [13]. Based on the conducted studies, the combination of caffeic acid and erythromycin was selected for further testing.

Erythromycin is an antibiotic commonly used to treat staphylococcal infections, particularly in patients who are allergic to penicillin. Our previous study indicated the antibacterial action of caffeic acid and a combined effect of caffeic acid with erythromycin on staphylococcal strains [13]. Moreover, there is a report stating that caffeic acid inhibits the MrsA pump, which causes the efflux of erythromycin from bacterial cells using ATP energy [24]. The mechanism of caffeic acid antibacterial action is still not fully understood. There are several hypotheses explaining the antimicrobial effect of this compound, such as a change in bacterial cell membrane permeability or a disruption of cell membrane integrity [12]. Therefore, it is crucial to assess how caffeic acid interacts with erythromycin and determine its possible clinical benefits.

Despite growing interest in natural compound–antibiotic combinations, few studies have applied standardized methodologies for synergy assessment. Many natural compounds are evaluated solely based on MIC values, without further analysis such as IC_50_ determination or bacterial viability assays. Although several natural substances have demonstrated promising antimicrobial activity, research on their combined use with antibiotics remains limited—particularly when assessed using rigorous methods like the fractional inhibitory concentration (FIC) index. Moreover, comparative studies involving different staphylococcal species or clinically relevant strains are scarce, which limits the potential of the findings.

Caffeic acid, known for its antimicrobial and antioxidant properties, remains underexplored in this context. To address these gaps, our study investigates the antimicrobial activity of caffeic acid, both alone and in combination with erythromycin, using reference and clinical strains of S. aureus and S. epidermidis. We assess the MIC, IC_50_, bacterial viability, and potential synergistic effects using the FIC index.

## 2. Results

In the first stage of the study, the classification of examined clinical strains (*S. aureus* 1–4 and *S. epidermidis* 1–4) was performed using classical methods. The PCR-RFLP method was used to confirm the identification of the clinical strain. All isolates were successfully identified as belonging to the *S. aureus* or *S epidermidis* species, which ensured the reliability of subsequent analyses. Moreover, the phenotypic resistance profiles to methicillin and MLS_B_ antibiotics were marked for clinical isolates. Four *S. aureus* (from *S. aureus* 1 to *S. aureus* 4) and four *S. epidermidis* (from *S. epidermidis* 1 to *S. epidermidis* 4) clinical strains were selected for the study. From these isolates *S. aureus* 3, 4 and *S. epidermidis* 1, 2 showed resistance to methicillin and a constitutive mechanism of resistance to MLS_B_ antibiotics, while *S. aureus* 1, 2 and *S. epidermidis* 3, 4 remained susceptible to these antibiotics. Moreover, four reference strains (*S. aureus* ATCC 25923, *S. aureus* ATCC 43300, *S. epidermidis* ATCC 12228, *S. epidermidis* ATCC 35984) with known drug susceptibility profiles were also used in this study. The results of the drug susceptibility testing for methicillin and MLS_B_ antibiotics are presented in Table 1. The determined drug resistance profiles were used for statistical analysis when their influence on the antibacterial activity of caffeic acid and the combination “caffeic acid–erythromycin” was checked.

In the second stage of the study, a series of dilutions of caffeic acid and erythromycin were made for each strain to investigate the MIC_0_ values for these compounds. After obtaining the absorbance and averaging the values from three samples, the MIC_0_ of E and MIC_0_ of caffeic acid were determined. The MIC_0_ of caffeic acid was 1024 µg/mL for all tested strains except *S. aureus* 1, whose growth was inhibited at a concentration of 512 µg/mL, and *S. epidermidis* ATCC 35984, with an MIC equal 256 µg/mL. The MIC_0_ values for erythromycin ranged from 0.25 to 1024 µg/mL. Table 1 presents the MIC_0_ values of caffeic acid and erythromycin against the examined strains as well as the resistance profile of each isolate.

**Figure 2:**
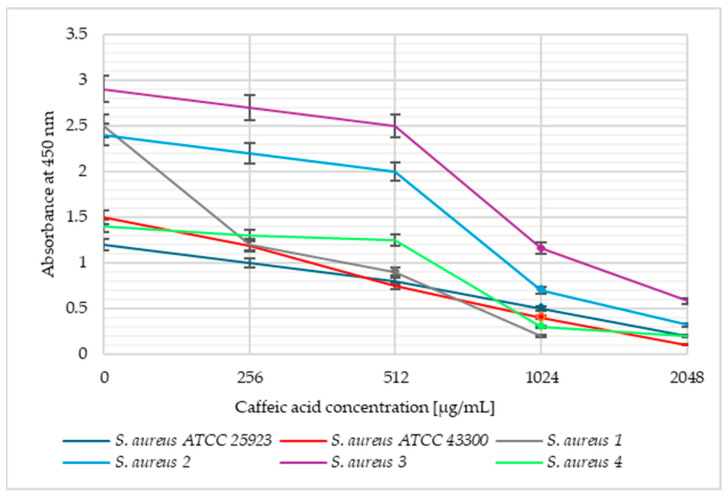
The plot of *Staphylococcus aureus* cell viability versus caffeic acid concentration. The markers indicate the MIC values of a given strain.

**Figure 3:**
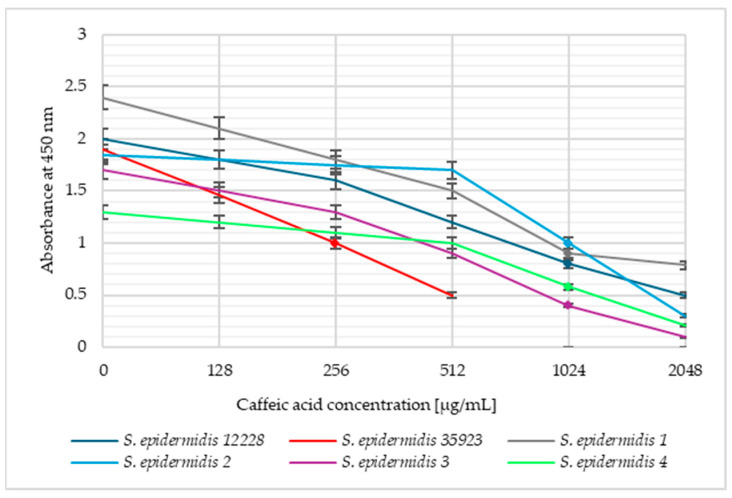
The plot of *Staphylococcus epidermidis* cell viability versus caffeic acid concentration. The markers indicate the MIC values of a given strain.

The viability of *S. aureus* and *S. epidermidis* bacterial cells was also determined at caffeic acid concentrations corresponding to 0.5 MIC, MIC, and 2 MIC. Figure 2 presents the absorbance versus the caffeic acid concentration for *S. aureus* and Figure 3 shows these dependencies for *S. epidermidis* strains.

The above graphs (Figure 2 and Figure 3) show that the viability of the tested strains in the same caffeic acid concentrations was slightly different and strain-dependent. As the concentration of caffeic acid increased, there was an increase in the reduction in bacterial viability. No significant differences in absorbance were observed for the two strains with lower MIC values (*S. aureus* 1 and *S. epidermidis* ATCC 35923) compared to other isolates. A slight decrease in absorbance was noted already at a concentration corresponding to 0.5 MIC. A significant reduction in viability was observed at concentrations corresponding to MIC values for each strain, but the strongest antibacterial effect was noted for 2 MIC.

**Figure 4:**
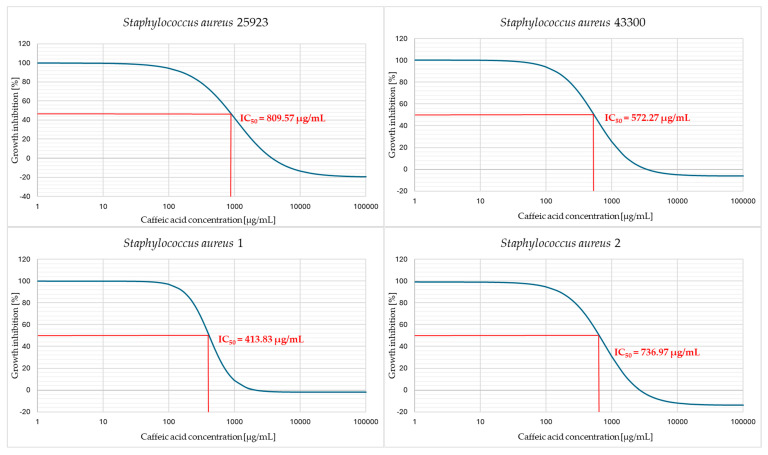

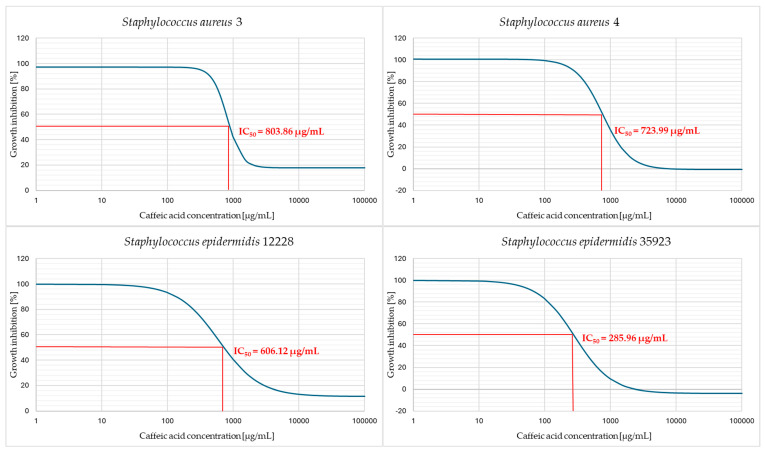

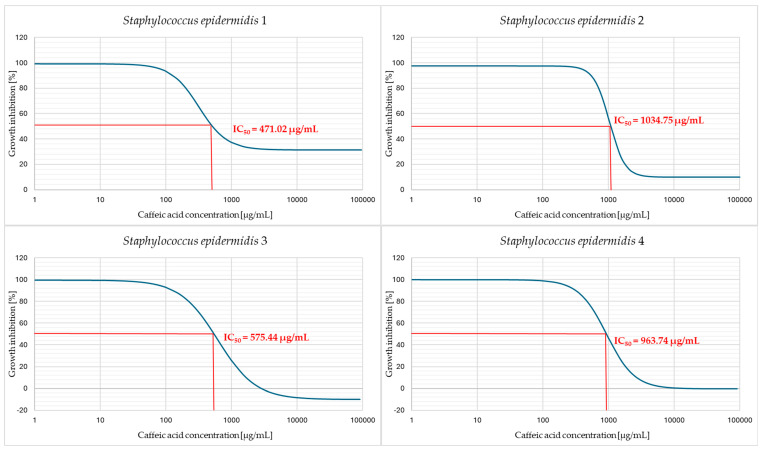
The relationship between the concentration of caffeic acid and the observed viability inhibition of *Staphylococcus aureus* and *Staphylococcus epidermidis* strains.

Then the IC_50_ values were determined by plotting the graphs of dependence of cell viability (expressed as a percentage of the control, which is considered 100%) versus caffeic acid concentration. Strains with the lowest MIC values also represented the lowest IC_50_ values. The highest IC_50_ value was recorded for the *S. epidermidis* 2 strain with a MIC of 1024 µg/mL. No significant differences in IC_50_ values were observed between *S. aureus* (average: 669.25 µg/mL) and *S. epidermidis* strains (average: 656.17 µg/mL). The methicillin and MLS_B_ resistance profile did not affect the IC_50_ values of caffeic acid on the tested strains (*p* = 0.57). Figure 4 shows the plot of the percentage bacterial growth inhibition versus caffeic acid concentration for each strain.

The next stage of the research was the checkerboard assay. The MIC_0_ values were used to construct a checkerboard. In the second stage of the study, the caffeic acid inhibited the growth of all the tested strains, with MIC values ranging from 128 to 2048 µg/mL. The most sensitive strain to caffeic acid was *S. epidermidis* ATCC 35984 with a MIC value of 128 µg/mL. Caffeic acid at a concentration of 256 µg/mL inhibited the growth of *S. aureus* 3. *S. epidermidis* 4 demonstrated MIC at 512 µg/mL. For *S. aureus* ATCC 25923, *S. aureus* ATCC 43300, *S. aureus* 1, *S. aureus* 4, *S. epidermidis* 1, and *S. epidermidis* 3, the MIC of caffeic acid was 1024 µg/mL. The highest MIC values at the level of 2048 µg/mL belonged to *S. aureus* 2, *S. epidermidis* ATCC 12228, and *S. epidermidis* 2.

The MIC values for erythromycin against the examined isolates ranged from 0.0313 to 2048 µg/mL. *S. epidermidis* 4 showed the lowest MIC value, 0.0313 µg/mL, while the highest (2048 µg/mL) was observed for *S. aureus* ATCC 43300, *S. aureus* 3, *S. aureus* 4, *S. epidermidis* ATCC 35984, *S. epidermidis* 1, and *S. epidermidis* 2. For *S. epidermidis* 3 the MIC of erythromycin was 0.125 µg/mL, and for rest of the tested strains, it was 0.25 µg/mL. The addition of caffeic acid resulted in a decrease in the MIC values of erythromycin for all tested strains, except *S. aureus* ATCC 25923, *S. epidermidis* ATCC 12228, *S. epidermidis* 1, *S. epidermidis* 3, and *S. epidermidis* 4.

Caffeic acid and erythromycin exerted a synergistic effect against *S. aureus* 2, *S. epidermidis* ATCC 35984, and *S. epidermidis* ATCC 35984. Additive interactions were observed against *S. aureus* 1 and *S. epidermidis* 1. The erythromycin–caffeic acid combination turned out to be indifferent against the rest of the examined strains.

The methicillin and MLS_B_ resistance profile did not affect the MIC values of caffeic acid on the tested strains (*p* = 0.30). Statistical analysis also revealed significant differences between MIC changes for resistant versus susceptible strains (*p* = 0.06).

**Figure 5:**
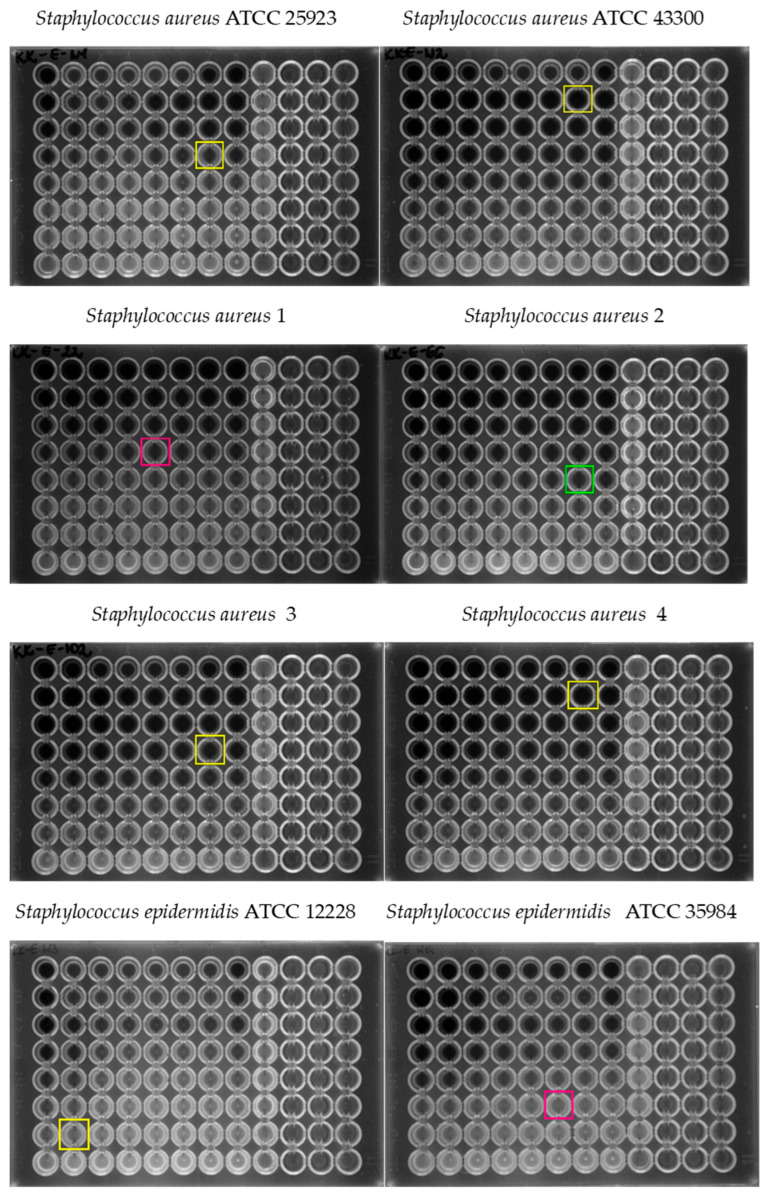

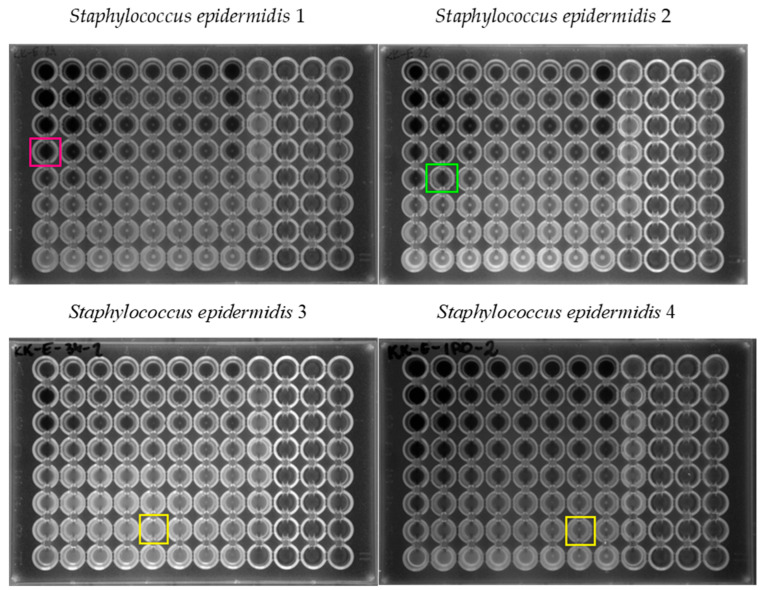
The checkerboard assay for staphylococcal strains. The FIC index for each strain is marked with a color. Yellow represents indifferent, pink represents additive, and green represents synergistic interactions. The FIC index was applied on the growth inhibition border and represents the point where the combinatory effect was the most visible.

The MIC values for both caffeic acid and erythromycin alone, MIC values for erythromycin with caffeic acid, and FIC index values are presented in Table 2. The results of the checkerboard test for each isolate are presented in Figure 5.

## 3. Discussion

Nowadays, nosocomial infections and the spread of drug resistance to various types of antibacterial agents among bacteria are a major public health problem. Most of the available antibiotics are inactive against many microorganisms which possess several resistance mechanisms. Therefore, it is highly important to look for new treatment regimens for multidrug-resistant strains and compounds which exert antibacterial potential. Such compounds may be substances of plant origin which could support the action of currently used antibiotics and increase their effectiveness in the fight against drug-resistant strains [6,9].

In our previous study (2018), the interactions of caffeic acid with antibiotics were investigated. Twenty-three strains of *S. aureus* were exposed to caffeic acid in combination with antibiotics, such as erythromycin, clindamycin, cefoxitin, and vancomycin, and we also examined the effect of caffeic acid alone. All tested strains showed sensitivity to caffeic acid, with their MIC values ranging from 256 to 1024 μg/mL. Therefore, the range of MIC values obtained was similar to those presented in this study. Moreover, in our previous study, no strain-dependent differences in MIC values compared to methicillin, and/or the MLS_B_ mechanism of resistance compared to strains without a mechanism, were observed. Among the interactions of caffeic acid with antibiotics, combined effects were observed in 16 strains of *S. aureus* treated with caffeic acid and erythromycin, while interactions of caffeic acid with the remaining antibiotics were observed in fewer strains. It should be noted that our previous work was a pilot study aimed at selecting the most promising caffeic acid–antibiotic combination for further research. Therefore, erythromycin was chosen for the studies presented in this manuscript. In the presented work, the methodology based on the determination of fractional inhibitory concentrations was used, which is the recommended method for testing the interactions between natural compounds and antibiotics, while our previous work was conducted using a different methodology. The MIC gradient test strips containing antibiotics were used to analyze the sensitivity of *S. aureus* to antimicrobial agents. The combined effects of CA and antibiotics were evaluated using MHA plates with the addition of a subinhibitory concentration of CA (one fourth of MIC CA). Comparing the obtained results of two reference strains that are repeated in both studies (*S. aureus* ATCC 25923 and *S. aureus* 43300) is therefore not entirely justified due to the different methodology [13].

The antimicrobial activity of various phenolic acids from Portuguese plants, including caffeic acid, was evaluated in Pinho et al.’s study. The tested microorganisms were, among others, *S. aureus* and *S. epidermidis.* For example, the MIC of caffeic acid against *S. aureus* ATCC 6538 and *S. epidermidis* ATCC 12228 was 625 µg/mL [25]. Compared to the MIC values of caffeic acid from the presented study (1024 and 2048 µg/mL, respectively), the reference strain *S. aureus* ATCC 6538 (which does not have the MLS_B_ antibiotic resistance mechanism) from Pinho et al.’s study showed greater sensitivity to caffeic acid than *S. aureus* ATCC 25923. Also, for *S. epidermidis* ATCC 12228, a higher MIC of caffeic acid, 2048 µg/mL, was noted in our study.

Pinho et al. also compared the activity of caffeic acid and other phenolic acids and reported that gallic acid had a stronger effect on all examined species. The MIC of gallic acid for *S. aureus* ATCC 6538 and *S. epidermidis* ATCC 12228 was 19.5 µg/mL and 9.8 µg/mL, respectively [25]. It is assumed that caffeic acid and gallic acid have the same mechanism of action, which is linked with the increase in bacterial cell membrane permeability. The greater antimicrobial activity of gallic acid may be due to its chemical structure. Gallic acid has an additional hydroxyl group connected to a benzene ring [25].

In addition to research on pure caffeic acid, extracts containing it are also often studied. The antibacterial activity of an aqueous extract of the *Thymus* plant against various strains of Gram-positive and Gram-negative bacteria was tested by Afonso et al. The *T. zygis* extract contained the highest amount of caffeic acid and it was the most active against the *S. aureus* strain (MIC = 1130 µg/mL) compared to all tested bacterial species. The obtained MIC value is comparable to the results from the present study. In turn, a much higher MIC value was recorded by Afonso et al. for *S. epidermidis* (4500 µg/mL) [26]. The antibacterial properties of *Citrullus colocynthis* extracts which contained caffeic acid were examined in Elansara et al.’s study. These extracts were more active against *S. aureus* than the caffeic acid alone tested in our study, since the MIC value of the methanolic extract was 190 µg/mL for *S. aureus* [27], while the MIC values of the pure compound against *S. aureus* in our work were 512–1024 µg/mL. The extract from the *Ocotea minarum* plant tested by Rodrigues et al., containing caffeic acid, also showed greater activity against *S. aureus* ATCC 25923 (500 µg/mL) [28].

It is worth mentioning that the above studies were not conducted using pure caffeic acid but on an extract containing this substance. This does not exclude the possibility of other active compounds contributing to its antimicrobial activity. Therefore, it is logical that their antibacterial activity is higher. Additionally, the studies suggest that Gram-positive bacteria are more susceptible to the action of caffeic acid than Gram-negative bacteria. This difference in susceptibility may be attributed to the structure of the cell membrane of Gram-negative bacteria, as their phospholipids can hinder the absorption of hydrophobic polyphenols [29].

The effect of the “caffeic acid–erythromycin” combination on staphylococcal strains has not been studied on a large scale so far, but below, the available literature data is discussed.

Erythromycin is a macrolide antibiotic, which acts by inhibiting protein biosynthesis by binding to the bacterial 50S ribosome subunit. In turn, the antibacterial mechanism of action of caffeic acid is related to increasing the bacterial cell membrane permeability [25,30,31]. Santos et al. evaluated the in vitro and in silico inhibition of *S. aureus* efflux pumps by caffeic and gallic acids. Caffeic acid presented the best results, effectively inhibiting the MrsA efflux pumps of *S. aureus,* which causes the efflux of erythromycin from bacterial cells. Caffeic acid also showed greater efficacy in the in silico model [24]. These studies suggest that the combined action of caffeic acid and erythromycin may have good therapeutic effects by inhibiting the mechanisms of bacterial resistance. Considering our previous studies and those of Santos et al., investigating the combination of caffeic acid and erythromycin seemed justified [13,24].

Considering the use of caffeic acid in therapy, its cytotoxic effect on human cells should be discussed. Phino et al. examined the cytotoxicity of caffeic acid towards human 3T3 fibroblasts. They proved that the effect of caffeic acid on cell viability was dose-dependent. Caffeic acid had no significant effect on cell growth at concentrations from 0.06 to 1.26 mg/mL. More than a 30% reduction in cell viability was noted after the use of concentrations of 6.31 mg/mL or higher. Caffeic acid can therefore be considered safe at concentrations lower than 6.31 mg/mL according to the above study [25]. Since the highest MIC of caffeic acid used in our experiment was 1.024 mg/mL, it can be concluded that the minimum concentrations that inhibit the growth of the tested staphylococci are safe.

There are only a few studies on the interaction of caffeic acid with erythromycin. The effects of various antibiotics and phytochemicals, including caffeic acid, on various strains of *S. aureus* were assessed by Kyaw et al. using the checkerboard method. Although their study did not evaluate the effect of caffeic acid in combination with erythromycin, the effect of interaction with other antibiotics, i.e., vancomycin, rifampicin, minocycline, ofloxacin, and cefotaxime, was examined. The study used the *S. aureus* ATCC 43300 reference strain, but clinical strains were also tested. For the reference strain an indifferent effect was noted, similarly to the present study [32]. The examined antibiotics are active against staphylococcal strains but have different mechanisms of action. Vancomycin inhibits cell wall synthesis, cefotaxime destroys the cell wall, and rifampicin and ofloxacin act at the level of DNA synthesis. Rifampicin is a DNA-dependent RNA polymerase inhibitor and ofloxacin is a gyrase inhibitor. Minocycline inhibits bacterial protein synthesis [32]. Erythromycin also inhibits protein synthesis. Since caffeic acid has an indifferent effect with all the antibiotics mentioned above, it can be concluded that the combined effect of caffeic acid with antibiotics depends more on the strain than on the mechanism of action of the antibiotic.

Natural compounds with MIC values higher than the MIC values of antibiotics cannot be used in monotherapy due to the insufficient antibacterial effect. However, a significant increase in the antibacterial properties of antibiotics in the presence of the natural compounds suggests that the synergistic effect they have in vitro may also increase the activity of antibiotics in vivo. Therefore, the field of application of caffeic acid includes the possibility of synergistic action in combination with antibiotics. Since, in the case of *S. aureus* strains isolated from wounds, significant decreases in erythromycin MIC values after the addition of caffeic acid were observed, and in the case of *S. epidermidis*, these decreases were much smaller, it seems that further research should focus on the local effects of caffeic acid. It could be used as an addition to dressings or ointments. Three recent studies complement our findings and broaden the understanding of caffeic acid’s utility in infection control. Zhang et al. conducted a study on a caffeic acid–cyclen–Zn(II) hydrogel, which demonstrated effective antibacterial and anti-inflammatory activity in a wound healing model, without cytotoxicity, highlighting the potential of CA-based wound treatments [33]. Similarly, Chiu et al.’s report described chitosan–caffeic acid membranes that significantly inhibited *S. aureus* and *Escherichia coli*. The authors suggested that these membranes inhibit the efflux pump of *Escherichia coli* [34]. Additionally, Jokubaite et al.’s study demonstrated that film dressings containing caffeic acid, prepared as controlled-release formulations, possess antimicrobial and antioxidant activity [35]. These examples reinforce our proposal to explore the local applications of caffeic acid (e.g., dressings or coatings) and support the hypothesis that its combination with erythromycin could enhance topical antibacterial effectiveness. However, since it showed additive and synergistic effects with erythromycin against strains isolated from blood and pericardial fluid, its systemic use, e.g., through the use of caffeic acid coatings on catheters, prostheses, etc., cannot be ruled out.

In the present study, caffeic acid and erythromycin were combined without the aim of synthesizing a new chemical compound, and the observed synergistic effect was likely due to pharmacodynamic synergy. Both compounds were applied separately but simultaneously in combination assays, and no chemical reaction between them was expected or observed. Therefore, they likely act as individual agents, potentially affecting different bacterial targets or modulating resistance mechanisms independently. However, as noted by Naqvi et al. [36], even minor structural modifications of existing antibacterial agents—such as fluoroquinolones—can result in significantly altered biological properties, including enhanced target specificity and improved cellular uptake. While their study primarily addressed fluoroquinolones as imaging agents, it underscores the broader principle that chemical interactions at the molecular level may open pathways to bypass resistance mechanisms. Although caffeic acid is a natural compound rather than a synthetic antibiotic, the same principle applies when evaluating its interaction with antibiotics like erythromycin at the molecular level, which could reveal potential synergies or novel modes of action that contribute to overcoming resistance in *Staphylococcus* strains. Therefore, future research should focus on precisely determining the mechanism of action of the caffeic acid on the bacterial cell and their cytotoxicity, which may contribute to the discovery of new strategies for the treatment of infections caused by multidrug-resistant staphylococcal strains.

In our study, the effect of caffeic acid on the staphylococcal cell viability was determined and, on this basis, the IC_50_ values for each strain were calculated. To the best of our knowledge, there have been no previous studies with this profile for caffeic acid against *S. aureus* and *S. epidermidis*. Although a reduction in viability was observed already at 0.5 MIC for each strain, a significant decrease in absorbance was noted for concentrations corresponding to MIC values and the strongest antibacterial effect was observed at 2 MIC. Since determination of the MIC value is more an observation than a calculation, checking the impact of a natural compound on the viability of bacterial cells at specific concentrations and determining the IC_50_ values seems to be more reliable and could significantly advance research based on the determination of the MIC value.

A limitation of the present study is the small group of tested strains. Moreover, a significant limitation of this research is the lack of cytotoxicity tests of caffeic acid on eukaryotic cells. This is a key aspect before considering the use of a new compound in patient-assisted therapy. Since *S. epidermidis* is a common cause of catheter or prosthesis infections due to its ability to form biofilms, another useful approach would be to evaluate the biofilm-inhibiting ability of caffeic acid. Future research should focus on all the above aspects and on determining the mechanism of action of caffeic acid on bacterial cells. Compared to the results of other research, some similarities can be observed. Natural compounds have a weaker effect on bacteria than antibiotics, but their combinations may be a good alternative in the era of increasing antibiotic resistance. The above studies also show that compounds such as caffeic acid or extracts of plants containing caffeic acid have a stronger antibacterial effect against Gram-positive bacteria. In addition, certain natural compounds in combination with another natural compounds may have stronger antimicrobial action compared to single agents. It is worth conducting more research in this direction to support the treatment of infections with antibiotic-resistant pathogens with natural compounds. Gram-positive bacteria were used in this study, but it is worth planning an experiment to check the interaction of caffeic acid with antibiotics on Gram-negative bacteria. Moreover, based on the studies mentioned above and the results presented in this paper, it seems justified to study combinations of caffeic acid with other antibiotics.

## 4. Materials and Methods

### 4.1. Materials

The study included six strains of *S. epidermidis* and six strains of *S. aureus*. There were four reference strains: *Staphylococcus epidermidis* ATCC 12228 and *Staphylococcus aureus* ATCC 25923 without the cMLSB resistance mechanism, and also *Staphylococcus epidermidis* ATCC 35984 and *Staphylococcus aureus* ATCC 43300 with the cMLSB resistance mechanism. Moreover, eight clinical strains of *Staphylococcus aureus* and *Staphylococcus epidermidis* were tested.

Clinical strains were obtained from microbiology laboratories of hospitals cooperating with the Department of Microbiology, Faculty of Pharmaceutical Sciences in Sosnowiec, Medical University of Silesia in Katowice, while ATCC strains were purchased (Sigma Chemical Co., St. Louis, MO, USA). All strains used in the study were archived in the Department of Microbiology, Faculty of Pharmaceutical Sciences in Sosnowiec, Medical University of Silesia in Katowice. *S. aureus* 1 was isolated from a wound swab. *S. aureus* 2 was isolated from a thigh wound. *S. aureus* 3 and 4 were isolated from a postoperative wound swab. *S. epidermidis* 1, 2, and 3 were isolated from blood, while *S. epidermidis* 4 was isolated from pericardial fluid. The exact characteristics of the tested clinical strains are presented in Table S1 in the Supplementary Materials and in Table 1 in the main paper. Before MIC and FIC determinations, all strains were cultured on blood agar at 37 °C for 24 h under aerobic conditions.

Caffeic acid was purchased from Sigma Chemical Co. (St. Louis, MO, USA) and dissolved in DMSO (Sigma Chemical Co., St. Louis, MO, USA) and water at a ratio of 1:5 (1 DMSO:5 water) before use.

### 4.2. Identification of Clinical Strains

The strains under investigation were first identified using standard microbiological methods such as hemolysis, catalase and coagulase tests, and anaerobic fermentation of mannitol. The API STAPH (bioMerieux, Marcy-l’Étoile, France) test was then used for further identification according to the manufacturer’s instructions. To confirm the identification of the clinical strains, the polymerase chain reaction–restriction fragment length polymorphism (PCR-RFLP) method was performed. Bacterial genomic DNA was isolated using the GeneMATRIX Tissue & Bacterial DNA Purification KIT (EuRx Ltd., Gdańsk, Poland), and a fragment of the *dnaJ* gene was amplified using specific primers (SA-(F) 5′-GCC AAA AGA GAC TATTAT GA-3′ and SA-(R) 5′-ATT GTT TAC CTG TTT GTG TAC C-3′). PCR reactions were performed based on the protocol provided by the manufacturer, considering the final volume—12.5 µL. The reaction mixture had the following proportions per sample: 1.25 µL of PCR RED, 0.1 µL of primer no. 1, 0.1 µL of primer no. 2, 10.55 µL of deionized water for PCR, and 0.5 µL of template DNA. To the reaction mixtures, 0.5 µL of DNA from the following standard strains was added: *S. aureus* ATCC 43300, *S. aureus* ATCC 25923, and *S. aureus* ATCC 6538. These were used as positive controls in individual PCR reactions. The negative control was the reaction mixture with 0.5 µL of deionized water. The PCR reactions were performed in the MJ Mini Personal Thermal Cycler by BIO-RAD. Then, to obtain species-specific restriction profiles, the PCR reaction products were treated with the restriction enzymes *XapI* and *Bsp143I* (Fermentas, Vilnius, Lithuania) by adding 5 µL of PCR product to 15 µL of a mixture containing 12 µL of water, 2 µL of buffer, and 1 µL of *XapI* or *Bsp143I* enzyme, which were then incubated for 3 h at 37 °C. The restriction fragments were separated by electrophoresis and their size was compared against a molecular weight marker [37].

### 4.3. Determination of the Resistance Profile of the Tested Clinical Strains

The antimicrobial susceptibility of the tested strains to macrolides, lincosamides, streptogramins B, and methicillin was assessed using the disk diffusion method, following the European Committee on Antimicrobial Susceptibility Testing (EUCAST) guidelines [38]. A commercial antibiotic disk (EMAPOL, Gdańsk, Poland) and Mueller–Hinton agar (MHA-BTL, Łódź, Poland) were utilized for these tests. A colony suspension equivalent to 0.5 McFarland unit (1–2 × 10^8^ CFU/mL) was inoculated onto Mueller–Hinton agar plates along with 2 μg clindamycin and 15 μg erythromycin disks to investigate the macrolide, lincosamide, and streptogramin B (MLS_B_) phenotype of resistance. The distance between the edges of the disks was maintained at 12–20 mm as per EUCAST recommendations. Methicillin-resistant phenotypes were determined using the disk diffusion method with a 30 µg cefoxitin disk. The zone diameter sizes were interpreted after 18 h of incubation at 35 °C, and strains were classified as resistant or sensitive based on the size and shape of the zone’s diameters.

### 4.4. Determination of the Minimal Inhibitory Concentration (MIC) Value for Erythromycin and Caffeic Acid

Erythromycin MIC_0_ and caffeic acid MIC_0_ values for each strain were determined by serial microdilution in a sterile 96-well polystyrene microtiter plate (FL Medical, Torreglia, Italy) with a final volume of 200 µL [39,40]. Stock solutions of caffeic acid and erythromycin were prepared from the powdered form of these substances. The MIC_0_ of caffeic acid for individual strains was determined in the concentration range from 2 to 2048 µg/mL, while the MIC_0_ of erythromycin ranged from 0.004 to 1024 µg/mL. All the samples were prepared and measured in triplicate. The microtiter plates were incubated in an incubator at 37 °C for 24 h under aerobic conditions. After incubation, bacterial growth was assessed by reading the optical density of individual microtiter plates at 595 nm using a MULTISCAN EX microplate reader (Thermo Electron Corp., Vantoa, Finland) [41,42]. Minimum inhibitory concentrations (MICs) were determined as the smallest concentration of the compound at which bacterial growth was completely stopped [43].

### 4.5. Determination of the Bacterial Cell Viability

The Kit-WST assay was used to determine bacterial cell viability according to the manufacturer’s instructions for Gram-positive bacteria. First, 10 µL of coloring reagent was added to the bacterial suspensions with caffeic acid after 18 h of incubation. The measurements were made three times for each strain and each concentration. Next, the microtiter plates were incubated at 37 °C. After incubation, absorbance was assessed by reading the optical density of individual microtiter plates at 450 nm using a MULTISCAN EX microplate reader (Thermo Electron Corp., Vantoa, Finland) [44].

### 4.6. Determination of the Half Maximal Inhibitory Concentration (IC_50_)

The IC_50_ of the caffeic acid against staphylococcal growth was determined by plotting the dose–effect curve (concentration versus the percent inhibition of bacterial cells) using nonlinear regression. Since the concentration values did not differ significantly from each other, a non-logarithmic scale was used. The IC_50_ was assumed to be the value at which cell viability was inhibited by 50% compared to the growth control. The percentage of bacterial-cell growth inhibition was determined according to the following formula:

%  =  100 (*Abs.control* − *Abs.sample*)/*Abs.control* (1)

where the *Abs.control* was a non-treated staphylococcal culture and *Abs.sample* was the absorbance of a single sample. The individual concentrations were tested for each strain in triplicate and each strain was tested three times. Then, the average of sample absorbance was calculated from the three measurements. Thus, the obtained IC_50_ values are an average of three repetitions and the obtained values are representative of three independent tests [45,46,47].

### 4.7. Determination of the Fractional Inhibitory Concentration (FIC) and FIC Index

The checkerboard microdilution method was used to determine the effect of the combined action of erythromycin and caffeic acid (FIC) [48,49]. The study was performed on a 96-well polystyrene microtiter plate (FL Medical, Torreglia, Italy) based on previously obtained values of MIC_0_ of erythromycin and MIC_0_ of caffeic acid for a *S. epidermidis* and *S. aureus* strains. In addition to FIC, the MIC of erythromycin and MIC of caffeic acid were also determined on the same plate for each isolate.

Seven concentrations of erythromycin and caffeic acid were prepared (8 MIC, 4 MIC, 2 MIC, MIC, 1/2 MIC, 1/4 MIC, 1/8 MIC), which were then added to appropriate wells (dilutions of erythromycin (50 μL to each well) were added successively to the wells of plate columns 1–7, and subsequent dilutions of caffeic acid (50 µL per each well) were added successively to rows A-G of the plate) to obtain the final concentrations of each of these compounds in order to determine the FIC (2 MIC, MIC, 1/2 MIC, 1/4 MIC, 1/8 MIC, 1/16 MIC, 1/32 MIC). In addition, 95 µL of liquid Mueller–Hinton medium and 5 µL of a bacterial suspension of each strain in physiological saline with a turbidity of 0.5 on the McFarland scale (1–2 × 10^8^ CFU/mL) were also added to each well, except to the background/sterility control wells. The microtiter plates were incubated in an incubator at 37 °C for 24 h under aerobic conditions. After incubation, bacterial growth was assessed by reading the absorbance of individual microtiter plates at 595 nm using a MULTISCAN EX microplate reader (Thermo Electron Corp., Vantoa, Finland).

FIC index values were calculated using the following formulas:

Erythromycin FIC = MIC of erythromycin with caffeic acid/Erythromycin MIC(2)

Caffeic acid FIC = MIC of caffeic acid with erythromycin/Caffeic acid MIC(3)

Fractional inhibitory concentration index (FICI) = FIC of erythromycin + FIC of caffeic acid(4)

Synergy was defined as FIC index ≤ 0.5; additive effect as 0.5 < FIC index ≤ 1; indifferent effect as 1 < FIC index ≤ 4; and antagonism as FICI > 4 [48,49].

### 4.8. Statistical Analysis

Statistical analysis of the change in erythromycin MIC values after the addition of caffeic acid depending on the presence or absence of the MLS_B_ or methicillin resistance mechanism in *S. epidermidis* and *S. aureus* strains was performed using the Mann–Whitney U test. Since the MIC_0_ values for erythromycin were measured in triplicate, the average of three calculations was considered in statistical analysis. The change in MIC value was defined as the difference between the average MIC of erythromycin and the MIC of erythromycin combined with caffeic acid.

The statistical analysis of the bacterial cell viability depending on the presence or absence of the MLS_B_ or methicillin resistance mechanism in *S. epidermidis* and *S. aureus* strains was also performed using the Mann–Whitney U test. The calculations of cell viability were performed three times for each strain and each concentration, and the mean values were used for statistical analysis.

Moreover, we checked whether there was a relationship between the MIC values of caffeic acid and the MLS_B_ or methicillin resistance mechanism of resistance among the examined strains using the Mann–Whitney U test. Since the MIC_0_ values for caffeic acid were calculated in triplicate, the average of three measurements was considered in statistical analysis.

Statistical analysis was performed using the Statistica 13.0 software, with a significance level set at *p* < 0.05.

## 5. Conclusions

Caffeic acid demonstrated antibacterial activity against *Staphylococcus epidermidis* and *Staphylococcus aureus* strains, and this effect was strain-dependent. Moreover, it reduced the bacterial cell viability at concentrations corresponding to MIC values, and the obtained IC_50_ values confirmed that it exerted antibacterial activity against staphylococcal strains. However, the cytotoxicity of caffeic acid at doses that minimally inhibit the growth of the tested strains should be tested before considering its implementation as an adjunct to therapy. Caffeic acid and erythromycin showed a synergistic effect when used together against three staphylococcal strains and an additive effect against two isolates. However, their combination was indifferent against the remaining seven strains. The change in the MIC values of the erythromycin after the addition of caffeic acid was not dependent on the MLS_B_ or methicillin resistance profile of the examined strains. Further studies should focus on determining the exact mechanism of action of the combination of caffeic acid and erythromycin. According to the literature data, the focus should be on the study of MsrA efflux pump inhibition and changes in bacterial cell membrane permeability.

## Figures and Tables

**Table 1:** The MIC_0_ values and resistance profile to MLS_B_ antibiotics and methicillin for *Staphylococcus aureus* and *Staphylococcus epidermidis*.

Tested Strain	MIC_0_ of Erythromycin (µg/mL)	MIC_0_ of Caffeic Acid (µg/mL)	Mechanism of Resistance to MLS_B_ Antibiotics	Mechanism of Resistance to Methicillin
*S. aureus* ATCC 25923	8	1024	-	MSSA
*S. aureus* ATCC 43300	1024	1024	cMLS_B_	MRSA
*S. aureus* 1	1	512	-	MSSA
*S. aureus* 2	1	1024	-	MSSA
*S. aureus* 3	1024	1024	cMLS_B_	MRSA
*S. aureus* 4	1024	1024	cMLS_B_	MRSA
*S. epidermidis* ATCC 12228	0.25	1024	-	MSSE
*S. epidermidis* ATCC 35984	1024	256	cMLS_B_	MRSE
*S. epidermidis* 1	1024	1024	cMLS_B_	MRSE
*S. epidermidis* 2	1024	1024	cMLS_B_	MRSE
*S. epidermidis* 3	1	1024	-	MSSE
*S. epidermidis* 4	1	1024	-	MSSE

cMLS_B_—constitutive mechanism of resistance to macrolides, lincosamides and streptogramin B; MSSA—methicillin-sensitive *Staphylococcus aureus*; MRSA—methicillin-resistant *Staphylococcus aureus*; MSSE—methicillin-sensitive *Staphylococcus epidermidis*; MRSE—methicillin-resistant *Staphylococcus epidermidis*.

**Table 2:** The MIC values for caffeic acid and erythromycin alone, MIC values for erythromycin with caffeic acid, and FIC index.

Tested Strain	MIC of Erythromycin (µg/mL)	MIC of Caffeic Acid (µg/mL)	MIC of Erythromycin with Caffeic Acid (µg/mL)	FIC Index	Interpretation
*S. aureus* ATCC 25923	0.25	1024	0.25	1.031	indifferent
*S. aureus* ATCC 43300	2048	1024	32	1.016	indifferent
*S. aureus* 1	0.25	1024	0.125	0.516	additive
*S. aureus* 2	0.25	2048	0.03125	0.141	synergistic
*S. aureus* 3	2048	256	32	1.016	indifferent
*S. aureus* 4	2048	1024	32	1.016	indifferent
*S. epidermidis* ATCC 12228	0.25	2048	0.25	1.016	indifferent
*S. epidermidis* ATCC 35984	2048	128	64	0.281	synergistic
*S. epidermidis* 1	2048	1024	2048	0.750	additive
*S. epidermidis* 2	2048	2048	1024	0.313	synergistic
*S. epidermidis* 3	0.125	1024	0.125	1.031	indifferent
*S. epidermidis* 4	0.0313	512	0.0313	1.063	indifferent

## Data Availability

The original contributions presented in this study are included in the article/Supplementary Material. Further inquiries can be directed to the corresponding author.

## Supplementary Materials

The following supporting information can be downloaded at: https://www.mdpi.com/article/10.3390/ph18070964/s1, Table S1: Characteristics of the tested *Staphylococcus aureus* and *Staphylococcus epidermidis* clinical strains.

## Abbreviations

The following abbreviations are used in this manuscript:

IC_50_	Half maximal inhibitory concentration
MIC	Minimum inhibitory concentration
FIC	Fractional Inhibitory Concentration
WHO	World Health Organization
PCR-RFLP	The polymerase chain reaction–restriction fragment length polymorphism
EUCAST	European Committee on Antimicrobial Susceptibility Testing
MLS_B_	Macrolides, lincosamides and streptogramin B
cMLS_B_	Constitutive mechanism of resistance to macrolides, lincosamides and streptogramin B
MSSA	Methicillin-sensitive *Staphylococcus aureus*
MRSA	Methicillin-resistant *Staphylococcus aureus*
MSSE	Methicillin-sensitive *Staphylococcus epidermidis*
MRSE	Methicillin-resistant *Staphylococcus epidermidis*

## References

[1] Nguyen T.H., Park M.D., Otto M. (2017). Host Response to *Staphylococcus epidermidis* Colonization and Infections. Front. Cell. Infect. Microbiol..

[2] Hatlen T.J., Miller L.G. (2021). Staphylococcal Skin and Soft Tissue Infections. Infect. Dis. Clin. N. Am..

[3] Gherardi G. (2023). *Staphylococcus aureus* Infection: Pathogenesis and Antimicrobial Resistance. Int. J. Mol. Sci..

[4] Murray C.J., Ikuta K.S., Sharara F., Swetschinski L., Robles Aguilar G., Gray A., Han C., Bisignano C., Rao P., Wool E. (2022). Global Burden of Bacterial Antimicrobial Resistance in 2019: A Systematic Analysis. Lancet.

[5] Burke Ó., Zeden M.S., O’Gara J.P. (2024). The Pathogenicity and Virulence of the Opportunistic Pathogen *Staphylococcus epidermidis*. Virulence.

[6] Ragheb M.N., Thomason M.K., Hsu C., Nugent P., Gage J., Samadpour A.N., Kariisa A., Merrikh C.N., Miller S.I., Sherman D.R. (2019). Inhibiting the Evolution of Antibiotic Resistance. Mol. Cell.

[7] Frieri M., Kumar K., Boutin A. (2017). Antibiotic Resistance. J. Infect. Public Health.

[8] Malczak I., Gajda A. (2023). Interactions of Naturally Occurring Compounds with Antimicrobials. J. Pharm. Anal..

[9] Al Alsheikh H.M., Sultan I., Kumar V., Rather I.A., Al-Sheikh H., Jan A.T., Haq Q.M.R. (2020). Plant-based Phytochemicals as Possible Alternative to Antibiotics in Combating Bacterial Drug Resistance. Antibiotics.

[10] Hamers V., Huguet C., Bourjot M., Urbain A. (2021). Antibacterial Compounds from Mushrooms: A Lead to Fight ESKAPEE Pathogenic Bacteria?. Planta Med..

[11] Muronetz V.I., Barinova K., Kudryavtseva S., Medvedeva M., Melnikova A., Sevostyanova I., Semenyuk P., Stroylova Y., Sova M. (2020). Natural and Synthetic Derivatives of Hydroxycinnamic Acid Modulating the Pathological Transformation of Amyloidogenic Proteins. Molecules.

[12] Catauro M., Barrino F., Poggetto G.D., Crescente G., Piccolella S., Pacifico S., Dal Poggetto G., Crescente G., Piccolella S., Pacifico S. (2020). New SiO_2_/Caffeic Acid Hybrid Materials: Synthesis. Materials.

[13] Kȩpa M., Miklasińska-Majdanik M., Wojtyczka R.D., Idzik D., Korzeniowski K., Smoleń-Dzirba J., Wasik T.J. (2018). Antimicrobial Potential of Caffeic Acid against *Staphylococcus aureus* Clinical Strains. Biomed Res. Int..

[14] Merlani M., Barbakadze V., Amiranashvili L., Gogilashvili L., Poroikov V., Petrou A., Geronikaki A., Ciric A., Glamoclija J., Sokovic M. (2019). New Caffeic Acid Derivatives as Antimicrobial Agents: Design, Synthesis, Evaluation and Docking. Curr. Top. Med. Chem..

[15] Sidoryk K., Jaromin A., Filipczak N., Cmoch P., Cybulski M. (2018). Synthesis and Antioxidant Activity of Caffeic Acid Derivatives. Molecules.

[16] Lukáč M., Slobodníková L., Mrva M., Dušeková A., Garajová M., Kello M., Šebová D., Pisárčik M., Kojnok M., Vrták A. (2024). Caffeic Acid Phosphanium Derivatives: Potential Selective Antitumor, Antimicrobial and Antiprotozoal Agents. Int. J. Mol. Sci..

[17] Ble-González E.A., Gómez-Rivera A., Zamilpa A., López-Rodríguez R., Lobato-García C.E., Álvarez-Fitz P., Gutierrez-Roman A.S., Perez-García M.D., Bugarin A., González-Cortazar M. (2022). Ellagitannin, Phenols, and Flavonoids as Antibacterials from *Acalypha arvensis* (Euphorbiaceae). Plants.

[18] Ben Mrid R., Bouchmaa N., Kabach I., Zouaoui Z., Chtibi H., El Maadoudi M., Kounnoun A., Cacciola F., El Majdoub Y.O., Mondello L. (2022). Leaves: A Valuable Source of Bioactive Compounds with Multiple Pharmacological Effects. Molecules.

[19] Nowak A., Cybulska K., Makuch E., Kucharski Ł., Różewicka-Czabańska M., Prowans P., Czapla N., Bargiel P., Petriczko J., Klimowicz A. (2021). In Vitro Human Skin Penetration, Antioxidant and Antimicrobial Activity of Ethanol-Water Extract of Fireweed (*Epilobium angustifolium* L.). Molecules.

[20] Mude H., Maroju P.A., Balapure A., Ganesan R., Ray Dutta J. (2022). Water-Soluble Caffeic Acid-Dopamine Acid-Base Complex Exhibits Enhanced Bactericidal, Antioxidant, and Anticancer Properties. Food Chem..

[21] Zhao X., Liu Z., Liu H., Guo J., Long S. (2022). Hybrid Molecules Based on Caffeic Acid as Potential Therapeutics: A Focused Review. Eur. J. Med. Chem..

[22] Khan F.A., Maalik A., Murtaza G. (2016). Inhibitory Mechanism against Oxidative Stress of Caffeic Acid. J. Food Drug Anal..

[23] Khan F., Bamunuarachchi N.I., Tabassum N., Kim Y.M. (2021). Caffeic Acid and Its Derivatives: Antimicrobial Drugs toward Microbial Pathogens. J. Agric. Food Chem..

[24] dos Santos J.F.S., Tintino S.R., de Freitas T.S., Campina F.F., Irwin I.R., Siqueira-Júnior J.P., Coutinho H.D.M., Cunha F.A.B. (2018). In Vitro e in Silico Evaluation of the Inhibition of *Staphylococcus aureus* Efflux Pumps by Caffeic and Gallic Acid. Comp. Immunol. Microbiol. Infect. Dis..

[25] Pinho E., Ferreira I.C.F.R., Barros L., Carvalho A.M., Soares G., Henriques M. (2014). Antibacterial Potential of Northeastern Portugal Wild Plant Extracts and Respective Phenolic Compounds. Biomed Res. Int..

[26] Afonso A.F., Pereira O.R., Válega M., Silva A.M.S., Cardoso S.M. (2018). Metabolites and Biological Activities of Thymus Zygis, Thymus Pulegioides, and Thymus Fragrantissimus Grown under Organic Cultivation. Molecules.

[27] Elansary H.O., Szopa A., Kubica P., Ekiert H., Ali H.M., Elshikh M.S., Abdel-Salam E.M., El-Esawi M., El-Ansary D.O. (2018). Bioactivities of Traditional Medicinal Plants in Alexandria. Evidence-based Complement. Altern. Med..

[28] Rodrigues A.B., De Almeida-Apolonio A.A., Alfredo T.M., Da Silva Dantas F.G., Campos J.F., Cardoso C.A.L., De Picoli Souza K., De Oliveira K.M.P. (2019). Chemical Composition, Antimicrobial Activity, and Antioxidant Activity of *Ocotea minarum* (Nees & Mart.) Mez. Oxid. Med. Cell. Longev..

[29] Duangjai A., Suphrom N., Wungrath J., Ontawong A., Nuengchamnong N., Yosboonruang A. (2016). Comparison of Antioxidant, Antimicrobial Activities and Chemical Profiles of Three Coffee (*Coffea arabica* L.) Pulp Aqueous Extracts. Integr. Med. Res..

[30] Xu P., Xu X.B., Khan A., Fotina T., Wang S.H. (2021). Antibiofilm Activity against *Staphylococcus aureus* and Content Analysis of Taraxacum Officinale Phenolic Extract. Pol. J. Vet. Sci..

[31] Luís Â., Silva F., Sousa S., Duarte A.P., Domingues F. (2014). Antistaphylococcal and biofilm inhibitory activities of gallic, caffeic, and chlorogenic acids. Biofouling.

[32] Kyaw B.M., Arora S., Lim C.S. (2012). Bactericidal Antibiotic-Phytochemical Combinations against Methicillin Resistant *Staphylococcus aureus*. Brazilian J. Microbiol..

[33] Zhang D., Gao W., Cui X., Qiao R., Li C. (2024). Caffeic Acid and Cyclen Based Hydrogel for Synergistic Antibacterial Therapy. ACS Appl. Mater. Interfaces.

[34] Chiu P.-H., Wu Z.-Y., Hsu C.-C., Chang Y.-C., Huang C.-M., Hu C.-T., Chang S.-C., Dai C.-A. (2024). Enhancement of antibacterial activity in electrospun fibrous membranes based on quaternized chitosan with caffeic acid and berberine chloride for wound dressing applications. RSC Adv..

[35] Jokubaite M., Ramanauskiene K. (2024). Potential Unlocking of Biological Activity of Caffeic Acid by Incorporation into Hydrophilic Gels. Gels.

[36] Naqvi S.A.R., Khan M.S., Iqbal J. (2017). Fluoroquinolones as Imaging Agents for Bacterial Infection. Dalton Trans..

[37] Shah M.M., Iihara H., Noda M., Song S.X., Nhung P.H., Ohkusu K., Kawamaura Y., Ezaki T. (2007). dnaJ gene sequence-based assay for species identification and phylogenetic grouping in the genus Staphylococcus. Int. J. Syst. Evol. Microbiol..

[38] The European Committee on Antimicrobial Susceptibility Testing Breakpoint Tables for Interpretation of MICs and Zone Diameters. Version 14.0, 2024. http://www.eucast.org.

[39] Amsterdam D., Loman V. (2005). Susceptibility Testing of Antimicrobials in Liquid Media. Antibiotics in Laboratory Medicine.

[40] European Committee for Antimicrobial Susceptibility Testing (EUCAST) of the European Society of Clinical Microbiology and Infectious Diseases (ESCMID) (2003). Determination of minimum inhibitory concentrations (MICs) of antibacterial agents by broth dilution. EUCAST discussion document E. dis 5.1. Clin. Microbiol. Infect..

[41] Cudic M., Condie B.A., Weiner D.J., Lysenko E.S., Xiang Z.Q., Insug O., Bulet P., Otvos L. (2002). Development of Novel Antibacterial Peptides That Kill Resistant Isolates. Peptides.

[42] Devienne K.F., Raddi M.S.G. (2002). Screening for Antimicrobial Activity of Natural Products Using a Microplate Photometer. Braz. J. Microbiol..

[43] European Committee for Antimicrobial Susceptibility Testing (EUCAST) of the European Society of Clinical Microbiology and Infectious Diseases (ESCMID) (2000). Terminology relating to methods for the determination of susceptibility of bacteria to antimicrobial agents. EUCAST definitive document E. Def 1.2. Clin. Microbiol. Infect..

[44] Tian L., Wang X., Zhang D., Wu M., Xue Z., Liu Z., Yang S., Li H., Gong G. (2021). Evaluation of the membrane damage mechanism of protocatechualdehyde against *Yersinia enterocolitica* and simulation of growth inhibition in pork. Food Chem..

[45] Sebaugh J.L. (2011). Guidelines for accurate EC50/IC50 estimation. Pharm Stat..

[46] Sorgi C.A., de Campos Chaves Lamarque G., Verri M.P., Nelson-Filho P., Faccioli L.H., Paula-Silva F.W.G. (2021). Multifaceted effect of caffeic acid against *Streptococcus mutans* infection: Microbicidal and immunomodulatory agent in macrophages. Arch Microbiol..

[47] “Quest Graph™ IC50 Calculator.” AAT Bioquest, Inc. 29 November 2024. https://www.aatbio.com/tools/ic50-calculator.

[48] Mazur P., Skiba-Kurek I., Mrowiec P., Karczewska E., Drożdż R. (2020). Synergistic ROS-Associated Antimicrobial Activity of Silver Nanoparticles and Gentamicin Against *Staphylococcus epidermidis*. IJN.

[49] Chai B., Jiang W., Hu M., Wu Y., Si H. (2019). In Vitro Synergistic Interactions of Protocatechuic Acid and Chlorogenic Acid in Combination with Antibiotics against Animal Pathogens. Synergy.

