# Supplementary material for: Caffeic Acid and Erythromycin: Antibacterial and Synergistic Effects on Staphylococci
Source: Pharmaceuticals (Basel). 2025 Jun 26;18(7):964. doi: 10.3390/ph18070964 (PMC12300647; doi:10.3390/ph18070964)
Supplement: Supplementary file 1 [file pharmaceuticals-18-00964-s001.zip › pharmaceuticals-3683139-supplementary.pdf]

Table S1. Characteristics of the tested *Staphylococcus aureus* and *Staphylococcus epidermidis* clinical strains.

Strain number	Hospital	Hospital Ward	Type of material collected for testing
S. aureus 1	Provincial Specialist Hospital of the Blessed Virgin Mary in Częstochowa	Orthopedic	Wound swab
S. aureus 2	Provincial Specialist Hospital of the Blessed Virgin Mary in Częstochowa	Pediatric	Thigh wound swab
S. aureus 3	Dr J. Daab Trauma Surgery Hospital in Piekary Śląskie	Spinal Injuries and Diseases	Swab from postoperative wound
S. aureus 4	Dr J. Daab Trauma Surgery Hospital in Piekary Śląskie	Septic	Swab from postoperative wound
S. epidermidis 1	Provincial Specialist Hospital No. 5 – Trauma Center in Sosnowiec	Intensive Care Unit	Blood
S. epidermidis 2	Provincial Specialist Hospital No. 5 – Trauma Center in Sosnowiec	Intensive Care Unit	Blood
S. epidermidis 3	Provincial Specialist Hospital No. 5 – Trauma Center in Sosnowiec	Intensive Care Unit	Blood
S. epidermidis 4	Independent Public Clinical Hospital No. 7 – Upper Silesian Medical Center named after Prof. Leszek Giec SUM in Katowice	Cardiac Surgery Department	Pericardial fluid
